# An in vivo neovascularization assay for screening regulators of angiogenesis and assessing their effects on pre-existing vessels

**DOI:** 10.1007/s10456-012-9287-8

**Published:** 2012-08-24

**Authors:** Witold W. Kilarski, Ludvig Petersson, Peder Fredlund Fuchs, Marcin S. Zielinski, Pär Gerwins

**Affiliations:** 1 Institute of Bioengineering and Swiss Institute of Experimental, Cancer Research (ISREC), École Polytechnique Fédérale de Lausanne (EPFL), Lausanne, 1015 Switzerland; 2 Department of Medical Biochemistry and Microbiology, Uppsala University, 751 23 Uppsala, Sweden; 3 Department of Radiology, Uppsala University, 751 85 Uppsala, Sweden; 4 Optics Laboratory, School of Engineering, Ecole Polytechnique Federale de Lausanne (EPFL), Uppsala, Sweden

**Keywords:** Angiogenesis, Animal model, In vivo, Chick chorioallantoic membrane, Drug screening, Wound healing, Tumor

## Abstract

Therapeutic regulation of tissue vascularization has appeared as an attractive approach to treat a number of human diseases. In vivo neovascularization assays that reflect physiological and pathological formation of neovessels are important in this effort. In this report we present an assay where the effects of activators and inhibitors of angiogenesis can be quantitatively and qualitatively measured. A provisional matrix composed of collagen I and fibrin was formed in a plastic cylinder and implanted onto the chick chorioallantoic membrane. A nylon mesh separated the implanted matrix from the underlying tissue to distinguish new from pre-existing vessels. Vascularization of the matrix in response to fibroblast growth factor-2 or platelet-derived growth factor-BB was scored in a double-blinded manner, or vessel density was measured using a semi-automated image analysis procedure. Thalidomide, fumagillin, U0126 and TGFβ inhibited neovessel growth while hydrocortisone exerted a negative and wortmannin a toxic effect on the pre-existing vasculature. This quantitative, inexpensive and rapid in vivo angiogenesis assay might be a valuable tool in screening and characterizing factors that influence wound or tumor induced vascularization and in assessing their effects on the normal vasculature.

**Electronic supplementary material:**

The online version of this article (doi:10.1007/s10456-012-9287-8) contains supplementary material, which is available to authorized users.

## Introduction

Expansion of the capillary bed by angiogenesis is in the healthy adult limited to wound healing, physiological growth of tissues (e.g. fat, skeletal muscle) and cyclic variations in the endometrium and the corpus luteum [2, 3]. Increased or aberrant angiogenesis is seen in a number of diseases such as tumor growth, proliferative retinopathies, rheumatoid arthritis and psoriasis [4–6]. Failure to restore proper organ function in conditions such as chronic wounds and in ischemic diseases of the heart or brain might be due to insufficient angiogenesis during tissue regeneration [7, 8]. Consequently, therapies that either inhibit or stimulate vascularization could offer new treatment options for a variety of diseases and both strategies have been explored in clinical trials [8–11]. A humanized anti-VEGF antibody has thus been approved for use in combination with standard chemotherapy for metastatic colon cancer, metastatic non-squamous non-small-cell lung cancer, metastatic renal cell carcinoma (RCC) and as monotherapy for recurrent glioblastoma multiforme [4, 12, 13]. However, other anti-angiogenic drug candidates showed no or limited effects on tumor growth in clinical trials despite promising effects in pre-clinical experiments e.g. the matrix metalloproteinase inhibitors marimastat [14] and batimastat [15], the shark cartilage extract Neovastat [16] and the α_v_β_3_ and α_v_β_5_ inhibitors S36578 and cilengitide [17, 18]. It is therefore important to acknowledge limitations in existing experimental models, including in vivo angiogenesis assays, in order to define cellular and molecular mechanisms that regulate tissue vascularization and to identify suitable therapeutic targets.

The optimal in vivo angiogenesis assay should permit absolute quantitative measurements of vascular ingrowth, give a clear distinction between newly formed and pre-existing vessels, allow non-invasive monitoring and be cost-effective, rapid, reproducible and reliable [19–23]. The growth of vessels during ontogenesis has been extensively studied using the chick chorioallantoic membrane (CAM) [24, 25], the mouse retina [26] and the zebra fish [27]. However, conclusions derived from developmental models cannot be directly translated into post-embryonic wound healing or tumor vascularization since these processes are driven by different mechanisms [28, 29]. Developmental vascularization is a genetically controlled process where vessels are formed de novo together with the surrounding tissue in a manner that is spatially and temporally reproducible. In contrast, neovascularization linked to different pathological conditions in the adult occurs in already differentiated tissues and is regulated by inflammation and less predictable liberation of local factors.

Frequently used non-developmental in vivo angiogenesis assays such as the CAM [24, 30–32], transparent chambers [33–35], matrix or sponge implants [22] and the cornea model [36, 37] all utilize the pre-existing vasculature as a source of vessels that expand into an implanted or injury-induced matrix. Even though these non-developmental in vivo models are more relevant for pre-clinical studies of pro- and anti-angiogenic substances, they do not allow discrimination of functionally perfused vessels from those with no or low perfusion when classical readouts such as counting vessels or proliferating endothelial cells on tissue sections, or measurement of total tissue blood content, are used [23, 38]. In addition, except for cornea and grid-based CAM models, none of these assays allow easy discrimination between new and pre-existing vessels.

The CAM assay is widely used for both developmental and post-developmental studies of angiogenesis due to easy access to the vascularized CAM membrane [24, 30, 39]. An additional advantage is that in many countries animal license is not needed for chicken embryo experimentation. The assay is rapid, inexpensive and suitable for large-scale screening of substances thought to regulate angiogenesis. However, the normal CAM tissue has a dense capillary network, which makes it difficult to distinguish new from pre-existing vessels when applying stimulatory substances directly on the CAM membrane [24, 25]. In addition, the membrane is sensitive to slight changes in pH, oxygen tension and physical or chemical injuries which results in a remodeling reaction with contraction of the CAM towards the irritant [24]. This creates a spoke-wheel pattern of radiating vessels that mistakenly might be interpreted as new vessels growing towards the stimulus [24, 32].

We have developed an angiogenesis assay that circumvents these shortcomings. A provisional gel-matrix of defined size and composition is placed on the CAM and vascularized in response to fibroblast growth factor-2 (FGF-2) or platelet derived growth factor-BB (PDGF-BB). The effects of pro- and anti-angiogenic substances on neovascularization as well as on pre-existing vessels can be determined separately as the matrix is separated from the CAM by a nylon mesh. Neovascularization is quantified either by binomial scoring or by a semi-automated image analysis procedure to detect more subtle effects. This in vivo model has the potential to become a valuable tool in large scale screening of substances developed with the aim to regulate angiogenesis.

## Materials and methods

### Preparation of the gel-holding chamber

A 0.5 cm high tube was cut from a plastic soft drink straw (5 mm in diameter) and glued with cyanoacrylate in the middle of a 1.5 × 1.5 cm square of a nylon mesh. Constructs were kept for at least 2 h at room temperature for the glue to harden. Constructs were then silanized for 2 min in Repel-Silane ES PlusOne (Pharmacia), washed once in isopropanol, twice in 99 % ethanol and twice in distillated water. Constructs were taken directly from water and placed on a paraffin film (Parafilm) that had been fixed in a humidified transparent plastic box. One box held two 15 cm long Parafilm strips and each strip carried two rows of 10 constructs. The chamber with 40 construct was incubated for 1 h at 50 °C which resulted in a weak fixation of constructs to the paraffin. Constructs were sterilized by spraying with 70 % ethanol, dried and kept at room temperature until preparation of the gel.

### Preparation of the fibrin-collagen implant

A 5× concentrated stock of 25 mg/ml fibrinogen was prepared by dissolving lyophilized bovine fibrinogen (Sigma) in a buffered solution of 107 mM NaCl, 2,700 IU/ml aprotinin, 250 mM HEPES pH 8.6 at 4 °C. The fibrinogen was dissolved by vortexing for 5 min and then filtered through a 0.45 μm sterile syringe filter, which required approximately one filter per 5 ml of fibrinogen stock. The fibrinogen was aliquoted, frozen and stored at −80 °C for no longer than 1 month. Collagen-fibrin gels were prepared by mixing one part of freshly thawed fibrinogen stock solution with 5 parts ice-cold rat-tail collagen (4 mg/ml, Upstate). The mixture was vortexed, quickly spun down to remove air bubbles and 100 μl of the mixture carefully pipetted into the gel-holding chambers. Gels were then incubated in a humidified box for 10 min at 37 °C to allow collagen to polymerize before addition of 50 μl of thrombin (2 IU/ml; Sigma) dissolved in clotting buffer (5 μM CaCl_2_, 10 μM MgCl_2_, 4.5 mg/ml streptomycin, 4.5 mg/ml penicillin, 11.25 mg/ml amphotericin B, 500 μg/ml BSA, 5,000 IU/ml aprotinin, 50 mM HEPES pH 7.6) and saline or 5 mg/ml FGF-2 (250 ng/construct). Where indicated FGF-2 was replaced with PDGF-BB (5 mg/ml, 250 ng/construct), VEGF (5 mg/ml, 250 ng/construct), or TGF-β (2 mg/ml, 100 ng/construct). After equilibration with the clotting buffer the theoretical pH of the gel was 7.6 and osmolarity 320 mOsM. Finally, 25 μl of sunflower oil was pipetted on top of the aqueous media. Oil prevented the gel from drying and infection during polymerization and incubation on the CAM and served as a solvent for fumagillin (32 μg), racemic mixture of thalidomide (2 mg), hydrocortisone (500 μg), U0126 (50 μg) or wortmannin (1 or 10 μg) as indicated. The amount of drug added to a construct was estimated from previously published in vivo experiments according to an empirical rule where the amount of drug per construct was three times higher than previously published amounts of drug/body weight/day. The reason for using this higher dose was that we only added the drug once and did not reiterate during the 6-day incubation period. The gels were allowed to polymerize over night at 37 °C in 95 % humidity. The next day gels were inspected and only constructs without gel leakage were kept for CAM implantation.

### Calculation of release curves of hydrophobic drugs

Constructs were prepared as described and 2 mg of fluorescein, tetramethylorhodamine or propidium iodide was mixed with 1 ml of sunflower oil and 25 μl of this mix was placed on the surface of the clotting buffer that covered the gel. Each construct was transferred to a well in a 24-well plate and 1 ml of Ringer solution was added to the bottom of the well. The plate was placed on a shaker at 37 °C and 25 μl samples of the Ringer were collected every 20–30 min and the absorbance of collected samples measured at the appropriate wavelength for each compound. The results were plotted on a time scale and linear regression slopes calculated. Data from two experiments were pooled and only the linear range of the plot was used to fit the linear regression.

### Gel implantation on the CAM

White Leghorn eggs were incubated at 38 °C in 70 % humidity for 3 days before the air sac was moved from the egg pole to the egg side by making a punch-hole in the air-sac pole and one hole in the side of the egg. The side hole was then taped and eggs were returned to the incubator for another 9 days. On day 12 of embryo development, the shell covering the air sac was taped to avoid potentially irritating shell fragments to fall down on the CAM while cutting a hole in the shell with scissors. A single construct was then placed on the CAM through the window in the shell and the opening immediately taped to avoid membrane drying and infection. Eggs were then returned to the incubator for additional 6 days and kept at 38 °C with 100 % relative humidity to avoid CAM drying.

### Visualization of neovessels

The whole eggs were fixed in 2× concentrated Zn-fixer (4.5 mM CaCl_2_, 51.5 mM ZnCl_2_, 32 mM Zn(CF_3_CO_2_)_2_, 1.5 mM Tris, 38.5 mM glycine, pH adjusted to 6.0; final osmolarity 340 mosmol/L) for 1 h and constructs then cut out from the CAM and the plastic tube was removed. This procedure left the fibrin/collagen gel, ingrown tissue with neovessels, and underlying pre-existing CAM attached to the nylon mesh. Fixation before tissue manipulation minimizes artifacts due to tissue injury and preserves the blood content within neovessels that can later be stained for hemoglobin. Vessels with functional circulation were identified by an intravascular injection of ink before Zn fixation. To access a vessel for ink injection a small crack was created and shell fragments carefully peeled off leaving the pergamon membrane intact. Applying a drop of sunflower oil increased the transparency of the pergamon membrane and revealed larger vessels in the CAM and made it possible to inject a CAM vein with 0.5 ml of colloidal carbon (India ink). To visualize all blood-containing structures, tissues were treated with a diaminobenzidine (DAB) solution (15 mM potassium acetate pH 5.0, 70 % ethanol, 40 mg/ml DAB, 0.3 % H_2_O_2_) that permanently stained hemoglobin and gave erythrocytes a red or brown color that persisted during subsequent treatment with benzyl benzoate/benzyl alcohol (BBBA; 1:1). Since DAB cannot diffuse freely through the thick gel or tissue mass (not shown), the remaining gel was partially lifted to allow influx of DAB under the gel to come in direct contact with ingrown vessels that were partially covered by the gel implant. This staining protocol allowed us to discriminate actively perfused vessels (ink perfused black vessels) from those with low or no perfusion (DAB stained red or brown structures). The CAM attached to the mesh underlying the implant was then photographed to document effects of drugs on pre-existing vessels. Thereafter the CAM was scraped off with a scalpel and the neovascular ingrowth on the opposite side of the mesh was photographed.

### Live imaging of vascular permeability

The fibrin/collagen gel was placed on the CAM and after 6 days of incubation the chicken was given an intravascular injection of TRITC-dextran (155 kDa; 100 μl of a 10 mg/ml solution). Vessels growing through the grid were identified and 10 μl of saline was added to this area. The neovasculature was imaged for 15 min with time-lapse microscopy at a frame rate of 1 image per second using a Leica M205 FA fluorescent stereomicroscope. Then 10 μl of human VEGF-A (200 ng/ml) was applied and imaging continued for additional 15 min.

### Quantitation and statistical analysis

Vascular ingrowth was scored on a binomial (yes or no) scale in a double-blinded manner. The results from independent experiments were pooled and differences between treatments were assessed using Fisher exact test with an α = 0.05 as significance level. To compare subtle differences in vascular ingrowth, vessel density was measured. Clarified (BBBA treated) implant tissues with the underlying CAM membrane removed were photographed (Nikon Coolpix 4500) with smallest (fixed) aperture using a magnification that covered the position of the original implant. Images (2,272 × 1,704 pixels) were saved as jpg with highest quality format and processed using Adobe Photoshop CS with IPTG 5.0 plugins. The process of analysis was standardized; resolution of images was converted to 300 dpi and vascular structures transformed into a one-pixel diameter skeleton representation. Vessel density was calculated by measuring total vessel length that was divided by the vascularized area. Hemorrhages and other interfering objects (e.g. shell fragments) were manually removed before image processing. Differences between treatments were tested with one-way ANOVA, followed by post hoc Tukey test for unequal sample sizes (Unequal N HSD test). For leakage analysis, GraphPad Prism was used to calculate if the slopes from the control are significantly different from the VEGF-A group.

## Results

### Preparation of defined and reproducible matrix implants

**Fig. 1:**
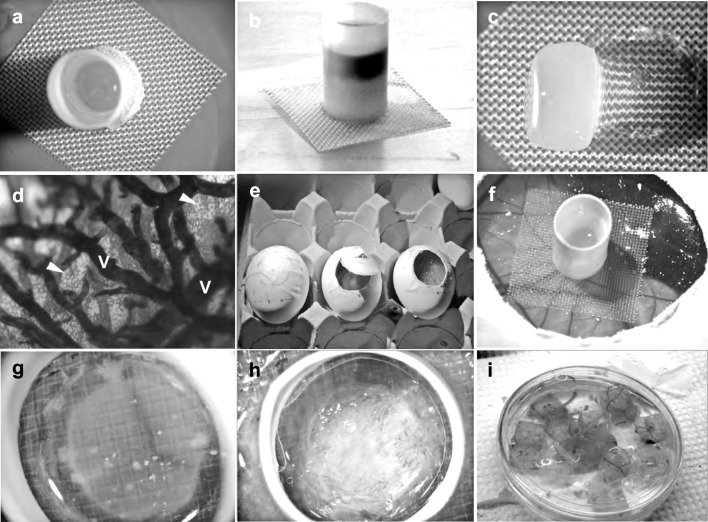
Technical outline of the implant vascularization assay. **a** A plastic straw was glued onto a nylon mesh and a fibrinogen-collagen solution applied in the straw. **b** The implant was covered with clotting buffer and plant oil. In this image the clotting buffer is stained black. **c** The polymerized gel does not stick to the silanized construct, which allowed the straw to be removed without affecting the gel. **d** Blood vessels of 12-days CAM were stained with DAB/H_2_O_2_. Superficial dense capillary network (*arrowheads*) is supported by larger feeding vessels (V). **e** The CAM membrane was exposed by cutting a window in the shell of White Leghorn eggs at day 12 of incubation. **f** A construct placed on the CAM. The nylon mesh separated the pre-existing CAM from the gel and distributed the weight over a larger tissue area. **g** Control and FGF-2 **h** containing constructs after 6 days of incubation. Parallel to vascular ingrowth gels were partially digested, contracted by invading cells and the gel opacity increased. **i** After whole-egg fixation, the constructs with the underlying CAM were excised and the plastic tubes detached

A matrix consisting of fibrin and rat-tail collagen type I was formed in a 0.5 cm high plastic tube that was glued onto a 1.5 × 1.5 cm piece of a nylon mesh. The tube gave gels a reproducible size and shape and allowed addition of substances expected to regulate vascularization (Fig. 1a–c). Application of a nylon mesh made it possible to distinguish neovessels growing through the mesh from pre-existing vessels (Fig. 1f). In addition, the mesh distributed the weight of the construct more evenly, which minimized mechanical damage to the CAM (Fig. 1f). Before forming the gel, the construct was silanized in order to create a non-sticky surface that allowed the gel to freely contract and change size during its vascularization on the CAM (Fig. 1g, h). Water-soluble compounds such as growth factors were mixed into the clotting buffer that was added on top of the polymerized gel (Fig. 1b). Finally, a layer of sunflower oil covered the clotting buffer in the construct, which created a closed and sterile environment that also prevented the gel from drying. The oil layer also served as a solvent for lipophilic substances that were released into the matrix during incubation on the CAM. A single gel construct was placed on the CAM at day 12 of embryo development through a window in the shell of a white Leghorn egg (Fig. 1e, f). After 6 days of incubation with the construct the entire egg was fixed in Zn fixer. The construct was then cut out from the CAM and the plastic tube removed. This left the fibrin/collagen gel, ingrown tissue with vessels, and the underlying pre-existing CAM tissue attached to the nylon mesh (Fig. 1i).

### Visualization of implant neovascularization in whole-mount preparation

**Fig. 2:**
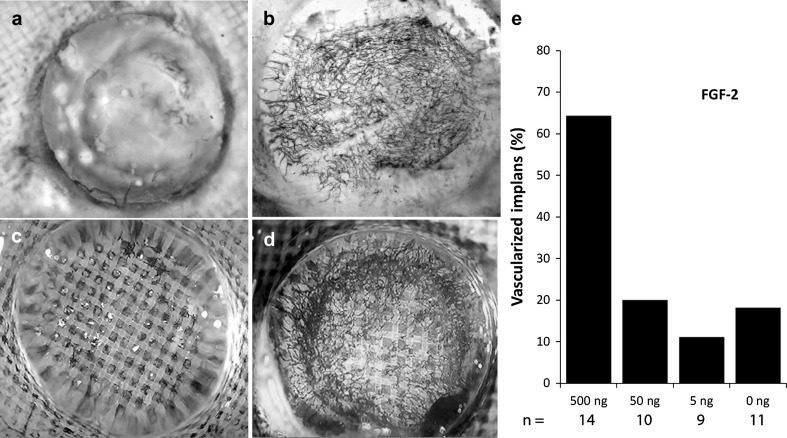
Visualization of neovessels and testing their functionality by erytrocyte staining and i.v. ink perfusion. **a** The remaining opaque gel covered the ingrown vasculature in samples that had been fixed and stained with DAB/H_2_O_2_. **b** Clarification with BBBA increased tissue and gel transparency revealing the ingrown DAB/H_2_O_2_ stained neovascular network. **c**, **d** To distinguish vessels with functional circulation from those with no or incomplete perfusion an intravenous injection of ink was performed before fixation and staining with DAB/H_2_O_2_. **c** Control and **d** FGF-2. The shape of transparent gel cap is visualized due to differences in refraction indices between air and BBBA. **e** Induction of neovascularization by FGF-2 is bipolar with no stimulation of vessels ingrowth at lower doses of FGF-2

The opacity of the gel increased during vascularization, which impaired detection of ingrown vessels (Fig. 2a). Blood vessels were therefore stained by treating CAM explants with a diaminobenzidine (DAB/H_2_O_2_) solution that permanently stained erythrocytes red (Fig. 2a, b). This vessel staining persisted after the tissue was treated with benzyl benzoate/benzyl alcohol (BBBA), which was used to render the opaque tissue transparent, and made it possible to visualize the entire neovasculature (Fig. 2b, d). Ink was injected intravenously to distinguish vessels with functional circulation from those filled with blood, but with no or incomplete perfusion. Ink is a non-diffusible colloidal stain that marks perfused vessels black while under- or non-perfused vessels remain red due to previous staining with DAB (Fig. 2b–d). Dose–response experiments indicated that FGF-2 dependent vessel ingrowth does not occur after reducing the amount of FGF-2 below a certain threshold. The number of inactive (red stained) vessels was negligible and restricted to morphologically aberrant structures e.g. locally enlarged neovessels or chemically injured pre-existing CAM vessels (Fig. 2d, compare with Fig. 5e) showing that nearly all ingrowing vascular structures were functional.

The optimal nylon mesh was selected by comparing mesh openings of 100, 300, 600 and 900 μm (not shown). The degree of vascular ingrowth was similar for all mesh sizes. However, the 100 μm mesh tended to bend with a non-uniform attachment to the CAM and the 600 and 900 μm mesh sizes often injured the CAM due to stiffer nylon threads. Therefore the 300 μm mesh size was selected.

### Implant neovascularization is enhanced by FGF-2 and PDGF-BB but not by VEGF-A

**Fig. 3:**
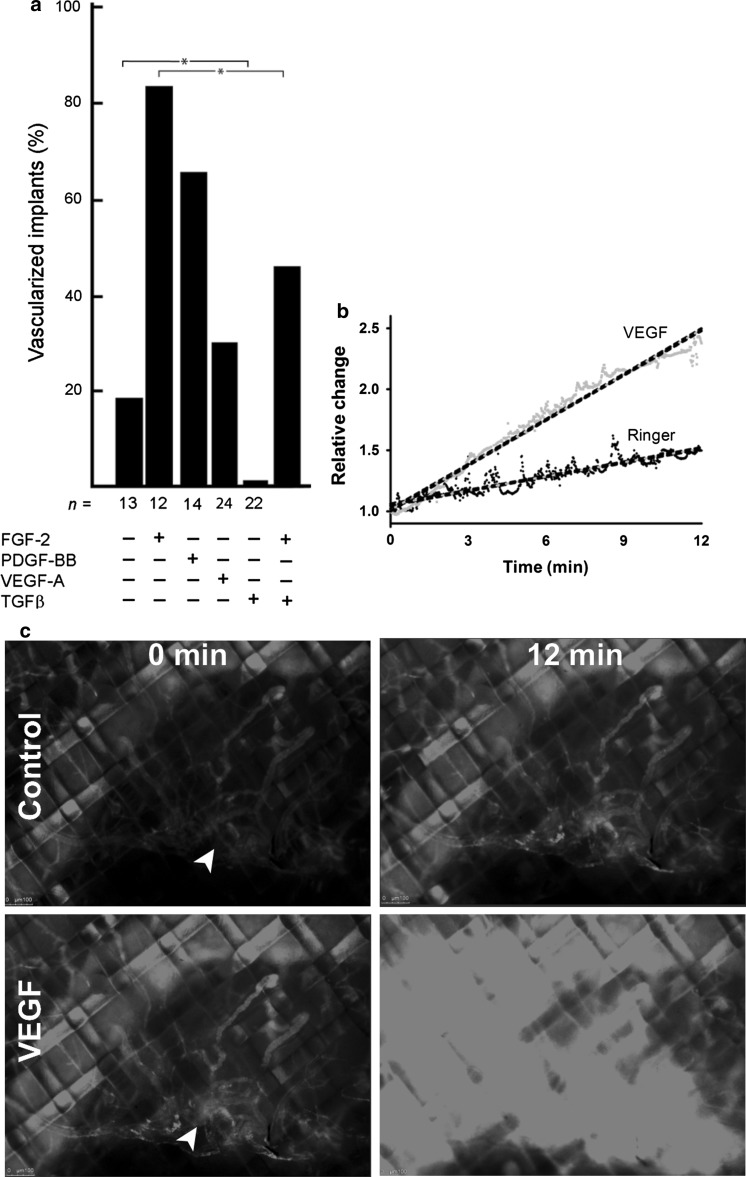
Effects of growth factors on neovascularization. **a** Growth factors were tested for their abilities to stimulate vascularization of a fibrin-collagen matrix implanted on the CAM and samples scored in a binomial manner (yes or no). FGF-2 (250 ng) and PDGF-BB (250 ng) induced vascularization, while human VEGF-A (250 ng) had no effect. TGF-β (100 ng) blocked spontaneous and reduced FGF-2-induced vessel ingrowth. **b**, **c** hVEGF-A induced TRITC-dextran extravasation from the implant neovasculature. 6 days after implantation 155 kDa TRITC-dextran was injected i.v. and neovessels that grew through the grid were first imaged for 15 min (12 min shown) after application of saline (control) and then for an additional 15 min (12 min shown) after stimulation with hVEGF-A (VEGF). Basal level of leakage from the neovessels was robustly increased after application of hVEGF-A. Note that in (**b**) the VEGF-A effect is underestimated as fluorescence signal quickly reached the saturation level (see Supplementary videos 1 and 2)

Implanted fibrin-collagen matrices were vascularized in 20 % of cases without addition of growth factors. FGF-2 or PDGF-BB caused a three to four fold increase in the number of vascularized implants. Surprisingly, VEGF-A [4] did not stimulate implant vascularization, while TGFβ had a strong inhibitory effect on spontaneous as well as FGF-2-induced neovascularization (Fig. 3a). The inability of human VEGF-A to induce neovascularization was not related to species differences as we found that hVEGF-A was recognized by chicken endothelium and induced a substantial increase in vascular permeability when applied on neovessels of the vascularized implant as shown by the TRITC-dextran extravasation assay, which is analogous to the Miles permeability assay [40] (Fig. 3b, c and Supplementary videos 1 and 2).

### Inhibition of angiogenesis can be distinguished from toxic effects on pre-existing vessels

**Fig. 4:**
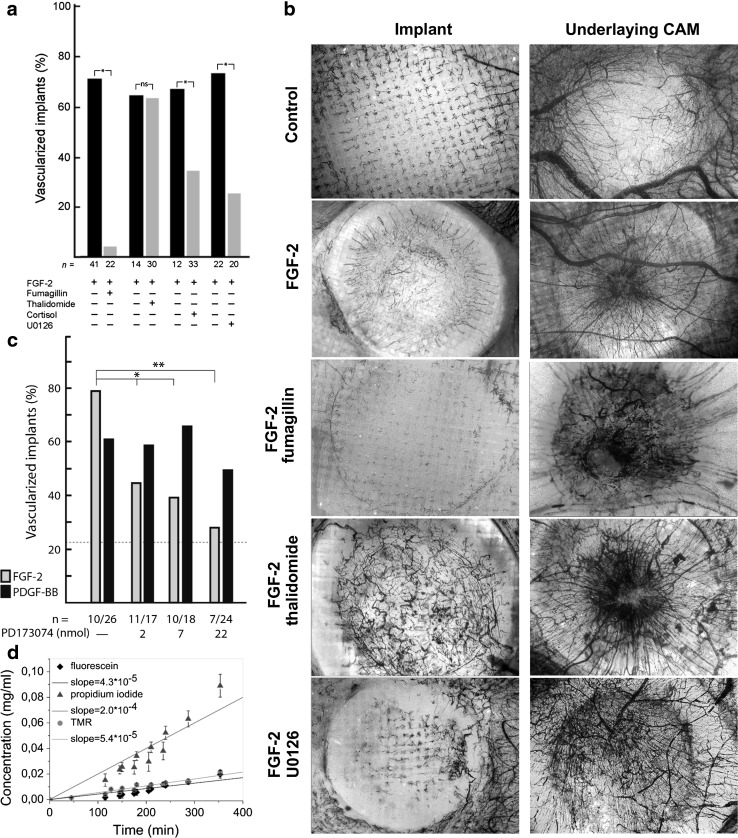
Effects of pro- and anti-angiogenic factors on neovessel ingrowth and on the pre-existing vasculature. **a** FGF-2 induced vascularization was significantly inhibited by fumagillin (32 μg), hydrocortisone (500 μg) and U0126 (50 μg) while thalidomide (2 mg) did not reduced the number of vascularized implants. *p < 0.05, *ns* non-significant. **b** Representative images of the effects of various treatments on implants vascularization and underlying CAM vasculature on. Gel implants with FGF-2 and angiogenesis inhibitors as indicated were applied on the CAM membrane and incubated for 6 days. Ink was injected intravenously prior fixation and samples were then clarified with BBBA. Pre-existing vessels in the CAM under the implant mesh were photographed and then scraped off to enable visualization of gel-ingrowing vessels. The “spoke-wheel” pattern in the CAM under the implanted mesh is the result of tissue contraction. **c** The FGFR1 and FGFR3 inhibitor PD173074 dose dependently inhibited FGF-2 but not PDGF-BB-induced neovascularization. **d** Release curves for hydrophobic (fluorescein and TMR) and water-soluble propidium iodide from oil. The average speed of release for fluorescein and TMR was 48.5 ng/min

**Fig. 5:**
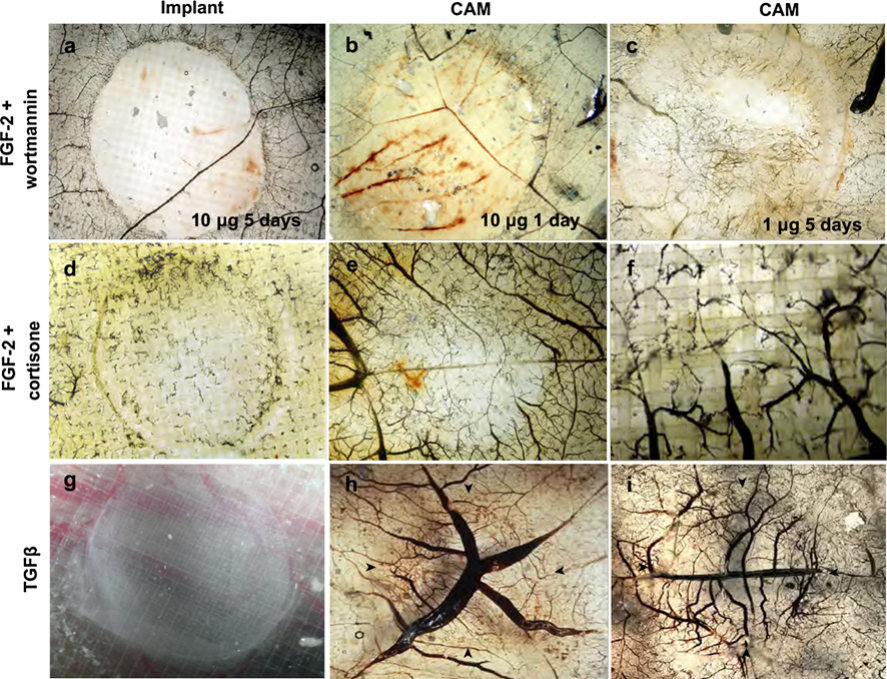
Effects of hydrocortisone, wortmannin and TGF-β on pre-existing vasculature. **a**–**c** Wortmannin (10 μg) blocked matrix vascularization (not shown) but also destroyed the pre-existing vascular network under the implanted gel (**a**). The toxic effect was apparent already 24 h (**b**) after implantation and was manifested by extensive hemorrhages and vessel occlusion (red vessels are not perfused with i.v. injected ink). It was not possible to distinguish toxic from inhibitory effects of wortmannin since 10-times lower dose (1 μg) was still toxic to pre-existing vessels (**f**), although the effect was limited to the central part of the CAM located under the implant. **d**–**f** Hydrocortisone (0.5 μg) inhibited neovascularization (**a**) and caused thinning of the pre-existing vessels in CAM underlying the implant without blocking vessel perfusion (**e**, **f**). **g**–**i** TGF-β (100 ng) inhibited spontaneous implant neovascularization (**g**) and caused enlargement of pre-existing vessels (**h**, **i**). *Arrowheads mark* the position of the gel. **a**–**f** vessels perfused with i.v. ink injection and stained with DAB/H_2_O_2_, **g**–**i** vessels only stained with DAB/H_2_O_2_

A selection of substances known to inhibit angiogenesis in vivo was tested for their ability to inhibit FGF-2 induced neovascularization of gel implants on the CAM. Both ingrowth of neovessels and potentially toxic effects of the drugs on pre-existing vessels in the CAM under the implants were analyzed (Fig. 4a, b). Vascular ingrowth was scored on a binomial (yes or no) scale in a double-blinded manner. Fumagillin [41] and the MEK inhibitor U0126 [42] inhibited neovascularization while thalidomide [43] had no effect on vessel growth using this method of quantification (Fig. 4a). None of these substances affected pre-existing vessels (Fig. 4b). PD173074, which is an FGFR1, FGFR3 and a weak VEGFR2 inhibitor, reduced FGF-2-dependent implant vascularization in a dose dependent manner but did not affect PDGF-dependent vessel growth. This experiment showed that an inhibitor added to the gel is released over time and that dose response relationships can be established (Fig. 4c). To further characterize release kinetics of hydrophobic substances from the oil phase we mixed the colored hydrophobic chemicals fluorescein and tetramethylorhodamin (TMR) (solubility in water for both is lower than 50 μg/ml) and water soluble propidium iodide (solubility in water 1–10 mg/ml) in oil and applied the oil mixture to the construct on the surface of the clotting buffer covering the gel. These chemicals have similar molecular masses as the hydrophobic inhibitors used in this study. The speed of release was calculated during the early linear phase with the assumption that the release of the drug from an implant on the CAM will not reach saturation as an excess of the drug is cleared from the tissue due to the circulating blood. Both hydrophobic compounds were released at similar speeds, while the water-soluble propidium iodide was released from oil at a 5 times higher rate. Assuming that the release of compounds from the oil phase is dependent on their water solubility the release speed of hydrophilic propidium iodide (200 ng/min) should constitute the upper limit for the speed of release for any water insoluble compound. The average value of the release slopes for fluorescein and TMR was 48.5 ng/min and we used this value to calculate approximate complete release times for compounds used in this study. For 2 mg of thalidomide the predicted release time was 670 h (25 days), for 32 μg of fumagillin 12 h, for 500 μg of hydrocortisone 180 h (7.5 days) and for 50 μg of U0126 18 h. Except for thalidomide all these compounds inhibited implant neovascularization even though fumagillin and U0126 should completely diffuse from the construct within the first day of incubation on CAM. Thalidomide was present in excess for the entire time of implant incubation on CAM but did not reduced vascularization using the yes/no scoring method. This was in contrast to the PI3K inhibitor wortmannin [44], which blocked matrix vascularization (Fig. 5a) but also exerted a toxic effect on normal CAM vessels causing vessel occlusion and hemorrhages already 24 h after implantation (Fig. 5b). Lowering the dose of wortmannin only reduced the area of the injury but had no effect on the mode of toxicity (Fig. 5c). Hydrocortisone [45] inhibited but did not block neovascularization (Figs. 4a, 5d) and caused thinning of vessels in the normal CAM vasculature but unlike wortmannin did not cause hemorrhages (Fig. 5e, f). Interestingly, transforming growth factor β (TGF-β) blocked spontaneous and FGF-2 induced implant vascularization (Figs. 3a, 5g) and also increased the diameters of the feeding CAM vessels located directly under the implant (Fig. 5h, i).

### Quantification of vessel density

**Fig. 6:**
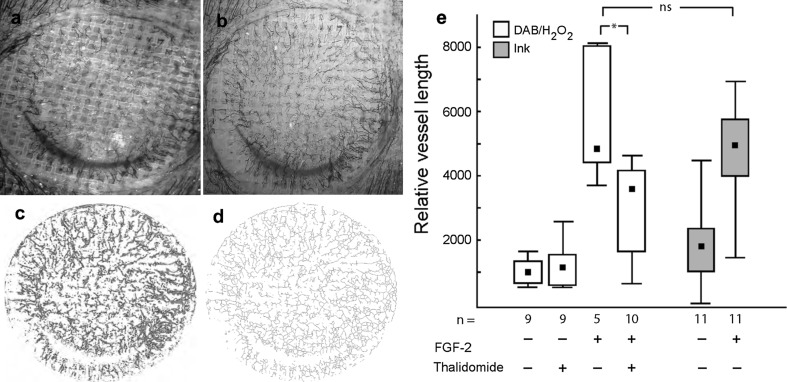
Semi-automated quantification of neovessel density. **a**–**d** Successive stages of image processing using Adobe Photoshop and IPTK plugins (Reindeer Graphics). Image resolution was set to 300 dpi and the raw image (**a**) was auto-leveled and sharpened (**b**) which was followed by edge enhancements (Stochastic edge plugin) from which surface iso-brightness lines were drawn (**c**). After dilatation by 3 pixels with the ranking filter, gray-scale branches were skeletonized (**d**) and the total skeleton length and its area were then calculated. **e** Thalidomide reduced neovessel density in FGF-2-stimulated implants. Different vessel staining techniques (DAB/H_2_O_2_ and i.v. ink injection) have no effect on calculated vessel density. Box-95 % percentile, whisker-Min-Max

To distinguish between different vascularization levels in the implant, we developed a technique to measure vessel density in the ingrown tissue. The calculated values reflect both the length and the density of neovessels. Successive stages of image processing are presented in Fig. 6a–d. By using this quantification method we found that thalidomide, which did not block neovascularization when vascularization was scored in a binomial manner (Fig. 4a) indeed reduced neovessel density in FGF-2 stimulated implants (*p* < 0.05; Fig. 6e). We also evaluated assay reproducibility by using two different methods of vessel detection with either erythrocyte staining (with DAB/H_2_O_2_) or by intravascular ink injection, which showed that assay variability was low as neither controls (*p* < 0.707) nor FGF-2-stimulated (*p* < 0.816) groups differ between independent experiments and different detection methods. These results confirm that blunt-ended and blood-filled vascular structures, either growing or regressing, can be neglected in the total vascular count.

## Discussion

Studies of angiogenesis are hampered by difficulties in distinguishing neovessels from pre-existing vessels, inability to discriminate inhibition of angiogenesis from vessel regression and problems related to quantification of the angiogenic response as well as determining the functionality of neovessels. In this report we describe an in vivo angiogenesis assay that circumvents these problems. We verified this model by testing known activators and inhibitors of angiogenesis with simultaneous assessment of their effects on pre-existing blood vessels. We have previously reported that the sequence of physiological events during neovessel ingrowth in this type of assay most closely reflects formation of vessels in granulation tissue during tissue healing with invasion of leukocytes, ingrowth of proto/myofibroblasts and formation of functional capillaries [31, 46].

The use of a nylon mesh to separate the pre-existing vasculature from neovessels entering the implant has previously been described [32, 47] and in this report we further expand the use of a mesh by combining it with a gel chamber which produces gel matrices with a reproducible size, shape and composition. The size of the mesh surface was increased 10 times as compared to the original publication by Nguyen [32] in order to distribute the weight of the implant over a larger surface area and to minimize mechanical damage to the CAM. The assay described in this report has several advantages as compared to other methods that use the CAM as a source of blood vessels for vascularization of an implanted matrix [32, 47, 48]. Since water-soluble factors are added to the gel after it has been polymerize in vitro, the matrix composition is consistent between different experiments and treatment groups. In vitro gel formation also reduces the variability of the final matrix that results from varying conditions during polymerization that occurs when matrices are formed directly on the CAM. Growth factors are, at least initially, protected from proteolytic degradation by addition of a high concentration of the serine protease inhibitor aprotinin. Application of plant oil as the last layer covering the gel and clotting buffer sealed the implant and protected it from drying and potential infections. Water-insoluble drugs could be dissolved in the oil phase which eliminated the need for using toxic organic solvents like DMSO. These organic solvents chemically damage the tissue which results in an artificial spoke-wheel pattern of vessels that can be misinterpreted as neovascular ingrowth (Supplementary Fig. 1). The use of a mesh made it possible to unambiguously distinguish ingrowing neovessels from the pre-existing vasculature, as vessels that appear above the grid by definition were neovessels. This is in contrast to e.g. the sponge implantation assay where distinction between new and pre-existing vessels is a recognized limitation [48].

The need for using of non-avian growth factors is a limitation for this and all other CAM based assays since mammalian proteins might not be recognized by the corresponding chicken receptors. Hence, the lack of an effect of human-derived cytokines has to be validated using other chicken models such as the ex vivo myofibroblast sprouting assay [49] or, as presented in this report, a chicken based vascular permeability assay. Since not all cytokines result in a biological response, the potential biological incompatibility of tested compounds constitutes a drawback of our model. The amount of growth factors per implant used in this study (250 ng/implant) is in the same range as used in similar assays e.g. 250–500 ng FGF-2 and 150 ng VEGF-A for gel implants on CAM [46, 47], 30 μg FGF-2 and 3 μg VEGF-A per implant in subcutaneous or cranial window assays in mice [50], 250 ng VEGF-A or FGF-2 per cornea pocket in rats [51], 200 ng of VEGF-A and FGF-2 on developmental CAM models [52] and 3.3 μg per implant of VEGF-A, PlGF-1, PlGF-2 and VEGF-C also using a developmental CAM assay [53]. Since 250 ng FGF-2 per construct induced implant vascularization and had no obvious adverse effect on animal well-being we decided to use this dose for testing the effects of other growth factors as they have similar dissociation constants (K_d_). Neovascularization in response to FGF-2 was binomial i.e. reducing the amount of FGF-2 from 500 to 50 ng per implant resulted in a complete lack of FGF-2-dependent neovascularization. We therefore decided to use a binomial yes/no scoring system for the initial quantification of the angiogenic response.

Visualization of the entire neovasculature is crucial for any in vivo angiogenesis assay. We describe a whole-mount staining protocol where erythrocytes were stained with DAB/H_2_O_2_ and the tissue is subsequently clarified with BBBA, which made it possible to visualize the entire neovasculature using a regular dissection stereomicroscope. The staining procedure is relatively fast, allowing quick binomial quantification of the angiogenic response and hence is suitable for large scale screening, in contrast to e.g. labor-intensive detection of endothelial markers in tissue sections. Combined injection of colloidal carbon and visualization of all blood containing structures by staining of hemoglobin was used to distinguish functional vessels from those with low or no perfusion. This provided a fast functional analysis of ingrowing vessels. Qualitative and quantitative analysis of double stained vascular ingrowth showed that a majority of neovessels were ink-perfused and thus properly integrated with the circulation. Live visualization of ingrowing neovessels can also be performed by fluorescent microscopy after an intravascular injection of TRITC-dextran. We show that this method can also be used to study changes in vascular permeability analogous to the Miles assay.

Quantification of neovascularization based on binomial scoring is sufficient for analyzing strong biological effects. If no differences between tested groups could be identified with the scoring method, a more labor intensive quantitative approach could be used where measurement of vessel density could detect subtle changes in the neovasculature. Only when there is no difference after quantification of vessel density that the conclusion “no inhibition” can safely be drawn when analyzing an angiogenesis inhibitor candidate. One example of this approach is thalidomide that only after measuring vessel density was revealed to have an anti-angiogenic effect. The quantification technique had low intra-experimental variation as the same groups (control vs. FGF-2) did not differ between experiments even though two different vessel-staining techniques were used.

The inability of VEGF-A to induce angiogenesis in this assay might have several explanations. One of the most profound effects of VEGF-A is increasing vascular permeability with extravasation of plasma and formation of a provisional fibrin matrix [40]. However, in the CAM model described in this report fibrin is already provided in the applied matrix. Furthermore, our assay was designed to study the isolated effect of a growth factor in the absence of tissue injury and a growth factor supplemented-provisional matrix is therefore applied on unharmed tissue. This is in contrast to other angiogenesis assays where surgery is used to apply VEGF-A like the pocket cornea assay, the skin chamber assay or the matrigel plug assay [21, 23]. Finally, the inability of VEGF-A to induce angiogenesis in CAM implants is in agreement with previous studies [47, 56].

The assay has a high capacity and we routinely performed two experiments per week with 240 eggs each. Experiments can be performed by a single trained technician and does not require sophisticated or expensive equipment. It is sufficient to have 15–20 embryos per experimental group in order to reach statistical significance if the effect is strong. Most of the times the binomial scoring system is sufficient, which makes quantitation simple and fast and results can be statistically tested (Fisher exact, χ^2^ test).

The assay described in this report permits parallel investigation of effects on neovascularization and on the pre-existing vasculature. Since toxic effects of pharmacological compounds on the normal vasculature might cause life threatening hemorrhages, this might provide valuable information during the pre-clinical testing phase. The angiogenesis inhibitor fumagillin and an inhibitor of the mitogen-activated protein kinase pathway (U0126) inhibited angiogenesis without affecting pre-existing vessels. Hydrocortisone has been shown to have an anti-angiogenic potential by affecting basal membrane composition [57] and was found here to reduce implant neovascularization and also caused thinning of pre-existing vessels. In contrast, the PI3K inhibitor wortmannin not only blocked implant vascularization but also had an immediate and strong toxic effect on normal CAM vessels causing hemorrhages and regression of vessels. The high toxicity on normal tissue most likely precludes the use of this compound in anti-angiogenic therapies. TGF-β did not induce implant neovascularization but blocked spontaneous and FGF-2-induced vessel ingrowth which is in agreement with previous studies [58]. In addition TGF-β caused enlargement of pre-existing macrovessels underlying the implant.

A number of pro- and anti-angiogenic molecules have entered clinical trials for a variety of diseases. However, in spite of promising results obtained from animal models, many therapies have failed when applied in the clinic. These discrepancies between bench and bedside suggest that current in vivo angiogenesis assays should be improved in order to better understand mechanisms of non-developmental angiogenesesis during tissue vascularization and repair. This in turn could aid in development of reagents that can specifically target neovascularization. The assay described in this report was developed in order to meet the suggested requirements for an optimal in vivo angiogenesis assay [19, 20, 22, 23].

## Electronic supplementary material

Below is the link to the electronic supplementary material.

Supplementary Figure 1 CAM wounding results in tissue contraction directed towards thesite of injury. (**a**) Live 13-day CAM with fragments of egg-shell marking the position of tissue. (**b**) Chemical wounding with topical application of 2 μl DMSO. The appearance of sharp macrovessels was a result of stasis in blood circulation. (**c**) 3 days after the chemical injury the surrounding tissue contracted towards the wound site as shown by relocation of egg shell fragments towards the wound center. Contraction also caused vessel bending towards the injury site (“spoke-wheel pattern”). (**d**) The vasculature was injected with ink, fixed and photographed from the CAM macrovessel side. (JPG 3169 KB)

Supplementary Video 1 At 6 days after implantation of the construct 155 kDa TRITC-dextran was injected i.v. CAM vessel and neovessels were imaged for 15 min (AVI 8625 KB).

Supplementary Video 2 After topical application of human VEGF-A imaging continued for additional 15 min (AVI 10239 kb)
