# Supplementary figures and images for: An in vivo neovascularization assay for screening regulators of angiogenesis and assessing their effects on pre-existing vessels
Source: Angiogenesis. 2012 Aug 24;15(4):643–55. doi: 10.1007/s10456-012-9287-8 (PMC3496524; doi:10.1007/s10456-012-9287-8)

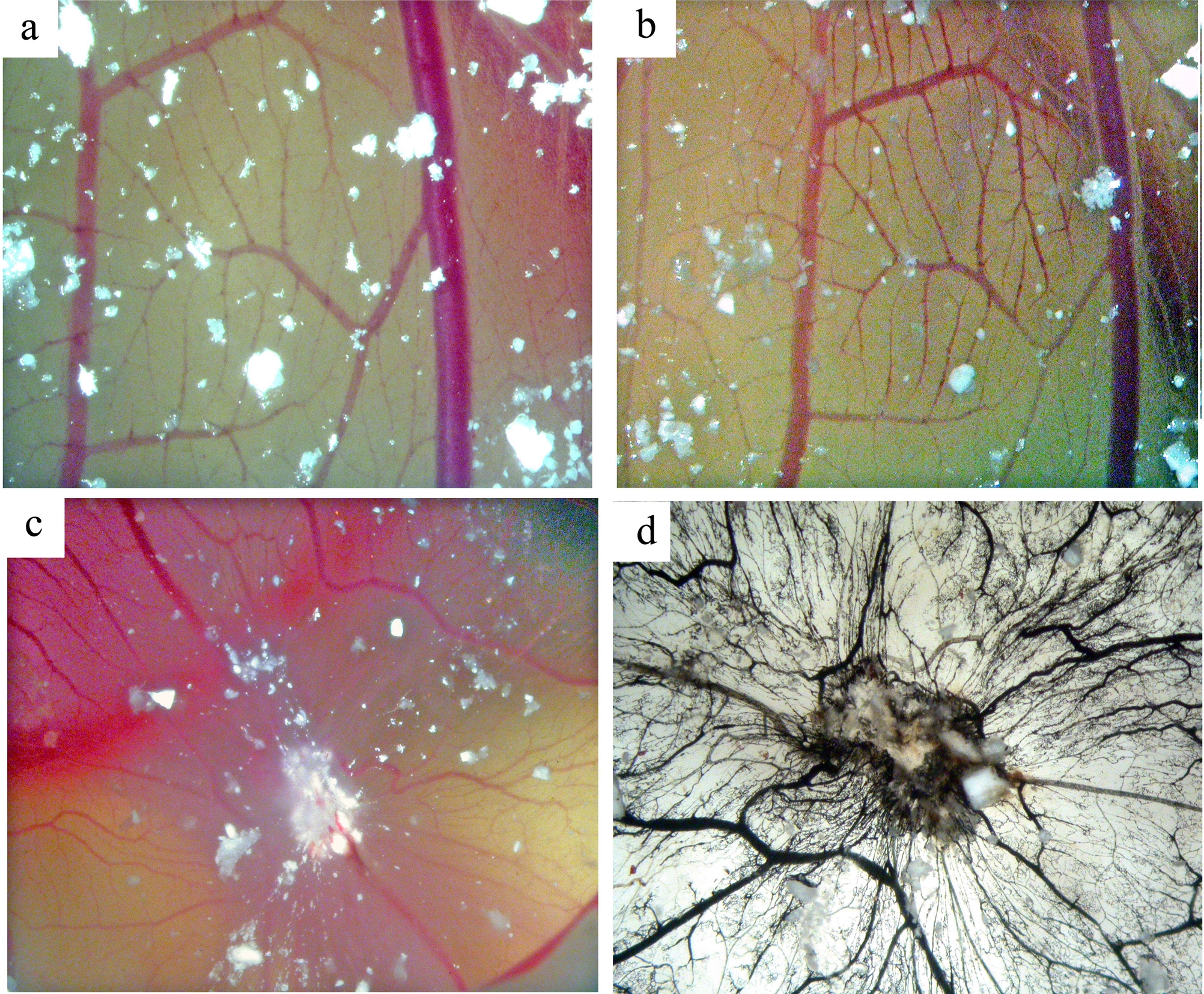

Supplement: Supplementary file 1 — CAM wounding results in tissue contraction directed towards thesite of injury. (a) Live 13-day CAM with fragments of egg-shell marking the position of tissue. (b) Chemical wounding with topical application of 2 μl DMSO. The appearance of sharp macrovessels was a result of stasis in blood circulation. (c) 3 days after the chemical injury the surrounding tissue contracted towards the wound site as shown by relocation of egg shell fragments towards the wound center. Contraction also caused vessel bending towards the injury site (“spoke-wheel pattern”). (d) The vasculature was injected with ink, fixed and photographed from the CAM macrovessel side. (JPG 3169 KB) [file 10456_2012_9287_MOESM1_ESM.jpg]
